# *In vitro* investigation of probiotic-mediated enhancement of soluble protein utilization and rumen fermentation efficiency

**DOI:** 10.3389/fvets.2026.1899102

**Published:** 2026-10-19

**Authors:** Xiaoqing Yang, Xiaoxue Ma, Ning Chen, Yayin Qi, Shu Li, Yanyan Wu, Wenju Zhang

**Affiliations:** 1 College of Animal Science and Technology, Shihezi University, Shihezi, China; 2 Xinjiang Agricultural Research Institute, Shihezi, China

**Keywords:** microorganisms, nitrogen utilization efficiency, probiotics, rumen fermentation, soluble protein

## Abstract

**Introduction:**

This study aimed to develop probiotics capable of efficiently utilizing soluble protein (SP) and to evaluate their effects on rumen fermentation and microbiota under different substrate protein structures.

**Methods:**

Cottonseed meal, corn flour, and wheat bran were fermented with seven single strains and combinations of probiotics. The selected compound probiotic, *Candida tropicalis* and *Enterococcus faecalis*, was then used to prepare substrates with three SP:insoluble protein (ISP) ratios (1:2, 1:2.5, and 1:3) for in vitro rumen fermentation. Fermentation parameters and the rumen microbial community were assessed.

**Results:**

*C. tropicalis*, Saccharomyces cerevisiae, and *E. faecalis* outperformed the other single strains in increasing substrate SP. The combination of *C. tropicalis* and *E. faecalis* significantly increased the substrate SP proportion. At an SP:ISP ratio of 1:2.5, total volatile fatty acids, acetate, propionate, and valerate were significantly increased. Crude protein degradation was significantly higher in the 1:2 and 1:2.5 groups than in the 1:3 group. The relative abundances of *Streptococcus* and *Ruminobacter* were significantly higher in the 1:2.5 group.

**Discussion:**

These in vitro findings provide preliminary evidence that optimizing the SP:ISP ratio in combination with compound probiotic supplementation may improve ruminal nitrogen utilization efficiency. Further in vivo validation is warranted.

## 1 Introduction

Probiotics are live microorganisms that confer health benefits to their hosts (1). They are widely used to enhance host immunity, regulate the balance of intestinal flora, and promote health (2). Probiotic fermentation can break down macromolecular proteins in feed into small peptides that are more easily absorbed and utilized by animals (3). During the fermentation process, probiotics rapidly proliferate under suitable conditions, competitively inhibit pathogens, suppress mold growth and the production of harmful metabolites in fermented feed, and thereby maintain intestinal health in animals (4). Previous studies support the notion of using compound microbial fermented feed in livestock and poultry farming (5). Fermented feed is rich in probiotics, organic acids, and functional metabolites, which can systematically enhance the antioxidant capacity, immune function, disease resistance, and production performance of animals through pathways such as optimizing the intestinal microbiota and strengthening the intestinal mucosal barrier function (6, 7).

Cottonseed meal (CSM), an abundant, locally sourced protein feed in the Xinjiang region of China, has an annual production reaching millions of tons. Further development and utilization of CSM can maximize the use of biological resources and effectively reduce feed costs (8). As a protein feed, the nutritional value of cottonseed meal depends largely on the degradation characteristics of its protein components in the rumen, and SP is a key factor affecting this process. During rumen fermentation, SP is rapidly utilized by rumen microorganisms (9). However, if the SP content in the diet is too low, it cannot provide sufficient nitrogen for rumen microorganisms to synthesize the microbial protein required for their growth. This limits the ability of microorganisms to decompose crude fibers, ultimately reducing the overall digestibility of the feed. Conversely, when the SP content in the feed is too high, ruminal protein may be degraded rapidly and excessively. This leads to excessive ammonia production, and the surplus nitrogen is excreted as urea. This not only wastes valuable protein resources but also increases the metabolic burden on the liver and kidneys following the absorption of excess ammonia, potentially causing toxicity (10). Traditional dietary formulations often suffer from problems such as an imbalanced protein supply, resulting in wasted feed resources, increased farming costs, and environmental pressure. Therefore, optimizing dietary protein structure and utilizing probiotic fermentation have become important areas in current ruminant nutrition research.

Although previous studies have investigated probiotic fermentation of protein feedstuffs and rumen fermentation separately, few studies have systematically combined these two approaches to manipulate the substrate protein structure as a quantitative variable. In particular, the effects of the SP:ISP ratio—a key determinant of ruminal nitrogen availability—on nitrogen utilization efficiency have rarely been evaluated in the context of compound-probiotic-fermented substrates. Moreover, most related studies have relied on single-strain fermentation or focused solely on feed quality improvement, whereas the present study integrates probiotic strain screening, quantitative manipulation of the substrate protein structure, and *in vitro* rumen fermentation to provide a comprehensive assessment of nitrogen metabolism and the associated microbial community. In addition, the use of locally sourced CSM as the major protein source confers practical relevance for the Xinjiang region.

On the basis of these considerations, we hypothesized that (1) compound probiotic fermentation would increase the proportion of SP in the substrate more effectively than single-strain fermentation, thereby providing a more readily available nitrogen source for rumen microorganisms; and (2) optimizing the substrate protein structure (i.e., the SP:ISP ratio) would further improve nitrogen utilization efficiency under *in vitro* rumen fermentation, as reflected in enhanced volatile fatty acid (VFA) production, substrate degradation, and the enrichment of protein-degrading microbial taxa.

To test these hypotheses, the present study aimed to (1) screen and prepare a compound probiotic capable of efficiently increasing SP content in substrates, using CSM, corn flour, and wheat bran as fermentation substrates; and (2) utilize *in vitro* rumen fermentation technology to investigate the effects of dietary substrates with different protein structures that have been fermented with the compound probiotic on rumen fermentation patterns, nitrogen metabolism efficiency, and the structure of the relevant microbial community. This study is expected to provide data support for the development of novel microecological feed additives based on local feed resources and preliminary guidance for the collaborative regulation of rumen nitrogen metabolism through dietary protein structure adjustment and probiotic supplementation.

## 2 Materials and methods

### 2.1 Animals and diets

Experimental procedures were approved by the Animal Ethics Committee of Shihezi University following a comprehensive review, approval number: A2025-1289. Three adult, healthy female Huaxi cattle (body weight: 574.98 ± 37.19 kg) from the animal facility of the Shihezi University Basic Experimental Station were selected as rumen fluid donors for the *in vitro* experiments. The feed formula and its corresponding nutritional indices, formulated according to the nutritional requirements for adult beef cattle, as stipulated by the National Research Council (NRC) (2012), are shown in Table 1. The cattle were fed equal amounts twice daily (morning and evening), had ad libitum access to water and were maintained under regular pen hygiene conditions.

**Table 1:** Ingredients and nutrient composition of the basal diet.

Item	Content
Ingredients, %	
Corn	16.01
Cottonseed meal	4.07
Soybean meal	12.18
Wheat bran	4.03
Alfalfa hay	15.75
Corn silage	27.75
Corn stalk	16.5
Magnesium oxide	0.44
Limestone	0.36
Sodium bicarbonate	0.44
Salt	0.44
Premix^1^	2.03
Total	100
Nutritional level^2^	
Crude protein (CP, % DM)	14.9
Ether extract (EE, % DM)	3.04
Neutral detergent fiber (NDF, % DM)	27.62
Acid detergent fiber (ADF, % DM)	13.47
Calcium (Ca, % DM)	0.63
Phosphorus (P, % DM)	0.27
Metabolizable Energy (ME, MJ/kg)	12.62

1. Per kilogram of premix contains: VD 320,000 IU, VE 3,000 IU, Fe 3,200 mg, VA 150,000 IU, Ca 130 g, P 30 mg, I 35 mg, Mn 1,500 mg, Cu 650 mg, Zn 2,000 mg, Co 10 mg, Se 10 mg. 2. Among the nutritional components, ME was the calculated value, while CP, EE, NDF, ADF, Ca, and P were measured; Nutrient levels were calculated based on dry matter (DM) basis.

### 2.2 Preparation of samples and inoculants

The ingredient samples selected for the experiment were CSM, corn flour, and wheat bran, which served as fermentation substrates (Table 2). Among them, CSM acts as the main nitrogen source to provide a high-protein substrate; corn flour serves as the carbon source to provide fermentable carbohydrates, which are necessary to maintain the carbon-nitrogen balance required for microbial growth; the loose and porous fibrous structure of wheat bran improves air permeability and water distribution within the substrate, thereby promoting oxygen transfer and supporting mycelial growth of aerobic microorganisms. Probiotic strains were preserved at the Biotechnology Laboratory of Shihezi University, including *Lactobacillus johnsonii*, *Streptococcus alactolyticus*, *Saccharomycopsis fibuligera*, *Saccharomyces boulardii*, *Candida tropicalis*, *Saccharomyces cerevisiae*, and *Enterococcus faecalis*, with a viable count of 1 × 10^8^ CFU/mL.

**Table 2:** Protein components of the raw material substrates.

Item	Protein components (Mean value)		
	CP (%)	SP (% CP)	ISP (% CP)
Wheat bran	16.3	34.6	65.4
Cottonseed meal	42.9	17.8	82.2
Corn flour	6.1	20.5	79.5

Values are means (*n* = 3). CP: crude protein; SP: soluble protein; ISP: insoluble protein. SP and ISP are expressed as percentages of CP.

The proteins in feed samples were fractionated in accordance with the CNCPS system (11). The crude protein (CP) content was determined via the Kjeldahl method (12). The soluble true protein (STP, corresponding to the PB1 fraction in the CNCPS) was directly quantified using the Coomassie Brilliant Blue (Bradford) assay (13). Non-protein nitrogen (NPN, corresponding to the PA fraction in the CNCPS) was measured by the trichloroacetic acid (TCA) precipitation method (14): true proteins were precipitated with TCA at a final concentration of 10%, and the nitrogen content in the resulting supernatant was subsequently determined. The ISP (corresponding to the combined PB2, PB3, and PC fractions in the CNCPS) was calculated using the formula: ISP = CP − NPN − STP. The SP content was calculated as the sum of NPN and STP, i.e., SP = NPN + STP.

### 2.3 Screening for single probiotics with high nitrogen utilization efficiency

For each group, 100 g of the substrate mixture (wheat bran: CSM: corn flour = 6:1:3, with SP accounting for 28.7% of CP) was prepared according to the formulation reported by Zhang (15), who demonstrated that a low-protein diet with SP accounting for 28.5% of CP enhanced nitrogen retention and net protein utilization. Seven different microbial suspensions (10^8^ CFU/mL) were added separately. The experiment was divided into eight groups: (A) *L. johnsonii*; (B) *S. alactolyticus*; (C) *S. fibuligera*; (D) *S. boulardii*; (E) *C. tropicalis*; (F) *S. cerevisiae*; (G) *E. faecalis*; and (H) blank (control). The substrate moisture content was set at 40%, and the inoculum volume was 8% (v/w). Each group comprised three replicates. The mixtures were then placed in conical flasks, sealed with gauze, and fermented for 48 h. After fermentation, the samples were dried at 45 °C, passed through a 40-mesh sieve, and stored for subsequent analysis. Evaluation indicators included SP, CP, microbial crude protein (MCP), ammonia nitrogen (NH_3_-N), and pH. The similarity sequence numbers between each treatment group and the reference sample for each indicator were calculated using DPS software (Version 9.01). A smaller sum of similarity sequence numbers indicates a higher comprehensive value for the treatment group. Based on this analysis, three probiotic strains with high SP utilization efficiencies were screened and selected.

### 2.4 Screening for compound probiotic combinations

Based on the three probiotic strains obtained from the screening described above, an orthogonal experimental design was employed to further optimize the conditions for the combination of *C. tropicalis* (E), *S. cerevisiae* (F), and *E. faecalis* (G). This was a 3-factor, 2-level optimization, in which each strain served as a factor with two levels (0 = not added, 1 = added); the factor-level coding is presented in Table 3. Four different combination schemes were derived according to the orthogonal array L_4_ (16), and the fermented substrates were evaluated for SP, CP, MCP, NH_3_-N, and pH. The optimal combination scheme was determined by range analysis: for each indicator, the range (R) of a factor was calculated as the absolute difference between the mean indicator values at its two levels, with a larger R indicating a greater influence of that factor. The combination that yielded higher SP, CP, and MCP and lower NH_3_-N and pH was then selected as the optimal compound probiotic combination with high SP utilization efficiency.

**Table 3:** Experimental factors and levels.

Level	Factors		
	*Candida tropicalis*	*Enterococcus faecalis*	*Saccharomyces cerevisiae*
1	0	0	0
2	1	1	1

“0” indicates the strain was not added, while “1” indicates the strain was added.

### 2.5 Preparation of samples with different substrate protein structures

Feed ingredients were used as substrate sources to formulate three substrates with different SP (% CP): ISP (% CP) ratios, designated as S1 (1:2), S2 (1:2.5), and S3 (1:3). The ratios were achieved by adjusting the proportions of the individual ingredients. Using the compound probiotic obtained from screening, the substrates were fermented under the same fermentation conditions as those described in section 2.3.

The rationale for selecting SP (%CP): ISP (%CP) ratios of 1:2, 1:2.5, and 1:3 was based on the following considerations: the three ratios correspond to SP proportions of approximately 33.5%, 28.7%, and 25.0% of CP, respectively. Previous *in vitro* studies demonstrated that when SP levels ranged from 30 to 40% of CP (with similar CP content of ~14%), total volatile fatty acids, acetate, propionate, and valerate concentrations were maximized with significant quadratic effects, whereas both lower (20%) and higher (50%) SP levels resulted in decreased dry matter digestibility and nitrogen digestibility (17). Furthermore, an *in vivo* feeding trial in fattening Hu sheep showed that higher nitrogen efficiency was obtained with an SP proportion of 25–30% of CP in low-protein diets (15). Davis (18) also reported that feeding dairy cattle a diet containing 15.5–16.5% CP and 30–35% SP (%CP, without urea) increased milk yield. Therefore, the SP range of 25.0–33.5% of CP in the present study was designed to encompass and further refine this previously identified optimal window. The three ratios were set at equal intervals (SP decreasing from ~33.5% to ~25.0% of CP) to enable systematic evaluation of the dose–response relationship between SP proportion and fermentation parameters, and all three fall within the range reported for commercial ruminant fattening diets (SP = 25–35% of CP). The ingredient composition and nutritional levels of the three substrates are listed in Table 4.

**Table 4:** Substrate ingredient composition and nutritional levels.

Item	S1	S2	S3
Ingredient composition, %			
Cottonseed meal	9	10	31
Corn flour	8	30	31
Wheat bran	83	60	38
Nutritional level			
Dry matter (DM, %)	88.9	88.4	88.6
CP(% DM)	18.2	17.1	24.3
SP(% CP)	33.5	28.7	25.0
ISP(% CP)	66.5	71.3	75.0

S1, S2, and S3 represent substrates with SP:ISP ratios of 1:2, 1:2.5, and 1:3, respectively. Nutritional levels are pre-fermentation values (dry matter basis). CP: crude protein; SP: soluble protein; ISP: insoluble protein.

### 2.6 Rumen fluid inoculation and *in vitro* cultivation

Rumen fluid was collected via the rumen fistula of beef cattle using a custom-made vacuum negative-pressure device before the morning feeding. The rumen fluid was filtered through four layers of cheesecloth into a pre-warmed thermos flask that had been prefilled with CO_2_ and maintained at 39 °C.

The artificial rumen buffer solution was prepared according to the method described by Wang (19) and Cone et al. (20). The buffer solution consisted of NaHCO_3_, NH_4_HCO_3_, Na_2_HPO_4_, KH_2_PO_4_, MgSO_4_·7H_2_O, Na_2_S, MnCl_2_·4H_2_O, CoCl_2_·6H_2_O, FeCl_2_·6H_2_O, CaCl_2_·2H_2_O, and resazurin dissolved in 1 L of distilled water. Prior to inoculation, the buffer solution was pre-warmed to 39 °C using a water bath and continuously gassed with high-purity CO_2_ until the pH reached 6.8 and the resazurin solution became colorless. The filtered rumen fluid was mixed with the buffer solution at a volume ratio of 1:2 under anaerobic conditions, and 30 mL of the mixture was dispensed into 100 mL glass syringes. A 0.2-g portion of each fermented substrate was placed into glass syringes and incubated in a constant temperature water bath at 39 °C for 48 h, with six replicates set for each group.

### 2.7 Determination of fermented feed parameters

The contents of SP and CP in the fermented feed were determined using the same methods as described in section 2.2.

The pH was determined by placing 5 g of fresh fermented product in a beaker to which 100 mL of ultrapure water was added. The mixture was allowed to stand at 4 °C for 24 h and then filtered into a conical flask. Five milliliters of the filtrate (extract) were used for pH measurements using a pH meter (pH 3,110 SET 2 Portable pH Meter WTW, Germany). The readings were recorded and the mean value was calculated. The processed extract was filtered through four layers of cheesecloth and stored in a − 20 °C freezer for subsequent determination of parameters such as NH_3_-N.

The NH_3_-N content in the fermentation samples was quantitatively analyzed using the phenol-sodium hypochlorite colorimetric method (HJ 536–2009).

The MCP content in the feed was determined using an Easy II Protein Quantitative Kit (TransGen Biotech Co., Ltd., Beijing, China).

### 2.8 Determination of fermentation parameters in the culture medium

Dry matter disappearance (DMD) was performed as follows: after fermentation was completed, the entire contents of each syringe were filtered through nylon bags (pore size: 20 μm). The residue was dried at 105 °C to constant weight to calculate DMD.

Crude protein disappearance (CPD) was performed as follows: the protein content in the residual substrate was measured using the Kjeldahl method, and subsequently used to calculate CPD.

The pH was measured using a portable pH meter (pH 3,110 SET 2 Portable pH Meter WTW, Germany).

The NH_3_-N content in the culture medium was quantitatively analyzed using the phenol-sodium hypochlorite colorimetric method (HJ 536–2009).

Volatile fatty acid (VFA) concentrations were determined by gas chromatography (Shimadzu GC-14B, Kyoto, Japan; capillary column CP-Wax 52 CB, 30 m × 0.53 mm × 1 μm; injector and detector temperature, 200 °C; initial column temperature 100 °C, increased to 150 °C at 3 °C/min; sensitivity set to 10^1^, attenuation at 2^5^) with some modifications. Briefly, the culture medium was thawed at 4 °C, centrifuged at 12,000 × *g* for 5 min, and 1 mL of the supernatant was taken. Subsequently, 0.2 mL of 20% metaphosphoric acid solution containing 60 mM crotonic acid (internal standard method) was added. The mixture was then filtered through a 0.22 μm syringe filter and analyzed using the gas chromatograph.

### 2.9 16S rRNA microbial flora sequencing analysis of the culture medium

The total genomic DNA of the microbial community from the rumen contents was extracted following the operating procedure of the E. Z. N. A.® Microbiome DNA Kit (Omega Bio-tek, Norcross, GA, U. S.). DNA quality was assessed by 1% agarose gel electrophoresis, and DNA concentration and purity were measured using NanoDrop 2000 (Thermo Fisher Scientific, Waltham, MA, USA). Subsequently, using the extracted DNA as template, the V3-V4 variable region of the 16S rRNA gene was amplified with the barcoded primers 338F (5’-ACTCCTACGGGAGGCAGCAG-3′) and 806R (5’-GGACTACHVGGGTWTCTAAT-3′).

PCR amplification was performed using TransStart FastPfu DNA Polymerase (TransGen, AP221-02). The reaction system was 20 μL, consisting of: 5 × FastPfu Buffer (4 μL), 2.5 mM dNTPs (2 μL), forward primer (5 μM, 0.8 μL), reverse primer (5 μM, 0.8 μL), FastPfu Polymerase (0.4 μL), BSA (0.2 μL), template DNA (10 ng), with the final volume brought up to 20 μL using ddH_2_O. The PCR program was set as follows: a. initial denaturation at 95 °C for 3 min (1 cycle); b. denaturation at 95 °C for 30 s, annealing at 55 °C for 30 s, and extension at 72 °C for 45 s (28 cycles); c. final extension at 72 °C for 10 min, followed by holding at 10 °C.

Subsequently, the PCR products were separated by 2% agarose gel electrophoresis, recovered, and purified using a DNA gel recovery and purification kit (PCR Clean-Up Kit, Yuhua, China), and the concentration of the recovered products was quantified using a Qubit 4.0 (Thermo Fisher Scientific). Libraries were constructed using the NEXTFLEX Rapid DNA-Seq Kit (Bioo Scientific, Austin, TX, USA). The main steps included: (1) adapter ligation; (2) magnetic bead-based screening to remove self-ligated adapter fragments; (3) PCR amplification to enrich the library templates; (4) recovery of PCR products using magnetic beads to obtain the final libraries. The final libraries were sequenced on the Illumina NextSeq 2000 platform (Shanghai Majorbio Bio-pharm Technology Co., Ltd.). Raw sequences were submitted to the NCBI Sequence Read Archive (SRA) (accession number PRJNA1404885).

### 2.10 Statistical analysis

All raw data were initially processed using Microsoft Excel 2016. Prior to parametric analysis, the normality of residuals was evaluated using the Shapiro–Wilk test, and the homogeneity of variances was assessed using Levene’s test. When these assumptions were not met, the data were transformed or analyzed using non-parametric tests (e.g., the Kruskal-Wallis test).

For the fermented feed parameters (SP, CP, NH_3_-N, MCP, and pH) and the single-endpoint culture medium parameters (VFA and CPD), differences among groups were tested by one-way analysis of variance (ANOVA) using IBM SPSS Statistics software (v22.0), followed by Duncan’s multiple-range test for *post hoc* comparisons. For the time-course culture medium parameters (pH, gas production (GP), NH_3_-N, and DMD), a two-way ANOVA was employed, with time, treatment, and their interactions as fixed effects and sample replicates as random effects; *post hoc* comparisons were performed using Tukey’s honestly significant difference (HSD) test. In all ANOVA analyses, each replicate sample within a group served as the experimental unit (*n* = 3 per group in the fermentation screening; *n* = 6 per group in the *in vitro* rumen incubation), and the significance level was set at *p* < 0.05. Figures were generated using GraphPad Prism software (version 10.1.2).

For the 16S rRNA gene sequencing data, alpha-diversity indices were compared among groups by one-way ANOVA, and *p*-values were adjusted for multiple testing using the Benjamini-Hochberg false discovery rate (FDR) correction. Beta-diversity differences were assessed by permutational multivariate analysis of variance (PERMANOVA; 999 permutations) based on Bray-Curtis distances. Differentially abundant taxa were identified by linear discriminant analysis effect size (LEfSe) analysis, with an LDA score threshold of 2.0, and the associated *p*-values were likewise FDR-corrected. The experimental unit for all sequencing analyses was the biological replicate sample from each incubation group (*n* = 6 per group).

The similarity priority ratio method in DPS software was applied to compare and rank the similarities between different probiotic treatments and a reference sample based on five indicators (SP, CP, MCP, NH_3_-N, and pH). Each test sample was assigned an identification code of 1, whereas the fixed reference sample was assigned an identification code of 0, and the “Fuzzy Similarity Priority Ratio Analysis” function was executed. The weights for the increase in SP, CP, and MCP contents and the decrease in NH_3_-N content and pH were set at 0.3, 0.2, 0.2, 0.2, and 0.1, respectively. A smaller sample sequence number indicates a higher degree of similarity to a fixed (reference) sample.

## 3 Results

### 3.1 Screening results of single probiotics

As shown in Figure 1, SP content in all groups (A-G) was significantly increased after substrate fermentation compared to that in the H group (blank control) (*p* < 0.05). Furthermore, the SP contents in groups E, F, and G were significantly higher than those in groups A-D (*p* < 0.05). Except for group A, the CP content in all treatment groups was significantly higher than that in the blank control (*p* < 0.05), with the highest CP contents observed in groups C and E, followed by groups D and F. MCP content was significantly increased in groups A, D, E, F, and G compared to that in the blank control (*p* < 0.05), whereas it was significantly decreased in group C (*p* < 0.05). No significant difference was observed between group B and the blank control (*p* > 0.05). Except for group E, the NH_3_-N content was significantly higher in all treatment groups compared to the blank control (*p* < 0.05). However, the NH_3_-N content in groups D-G was significantly lower than that in groups A-C (*p* < 0.05). Compared with the original substrate, the pH was significantly reduced in all groups (*p* < 0.05). In this experiment, the pH of all fermented substrates fell within the normal range of 4.0–5.5, consistent with typical microbial fermentation. In this trial, SP, CP, and MCP were considered positive indicators, NH_3_-N was a negative indicator, and pH was considered a range-based indicator. In summary, among the seven strains, *C. tropicalis*, *S. cerevisiae*, and *E. faecalis* demonstrated a significantly superior ability to increase substrate SP content and promote microbial protein synthesis.

**Figure 1:**
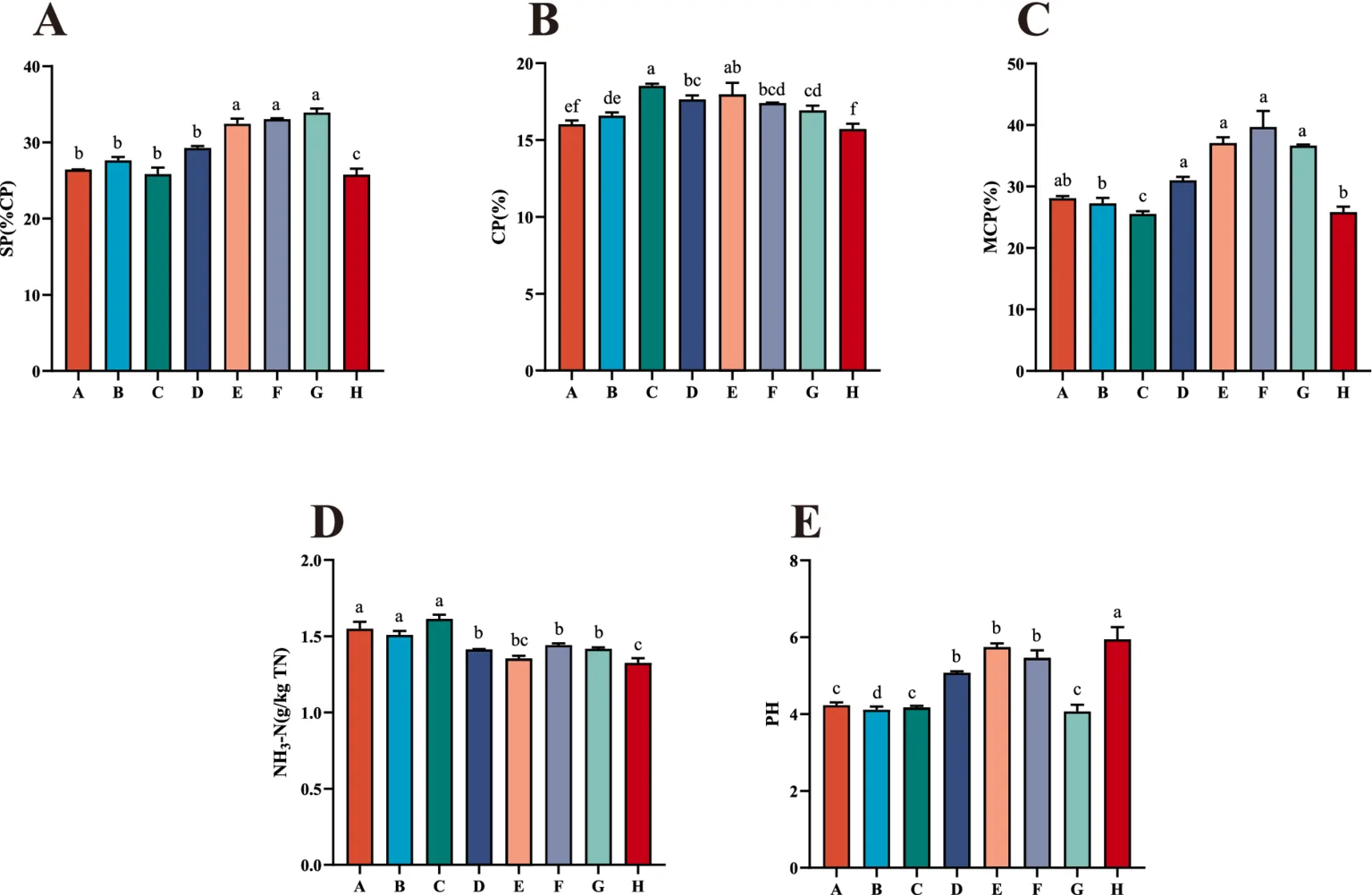
Measured values of single probiotic fermented feed (*n* = 3). SP: soluble protein **(A)**; CP: crude protein **(B)**; MCP: microbial crude protein **(C)**; NH_3_-N: ammonia nitrogen **(D)**; pH **(E)**. Groups A-G represent fermentation with *L. johnsonii*, *S. alactolyticus*, *S. fibuligera*, *S. boulardii*, *C. tropicalis*, *S. cerevisiae*, and *E. faecalis*, respectively; group H is the blank (non-inoculated) control. Data are presented as mean ± standard deviation (SD). Differences among groups were analyzed by one-way ANOVA followed by Duncan’s multiple-range test. Different lowercase letters within the same panel indicate significant differences (*p* < 0.05).

As shown in Table 5, the sum of the similarity sequence numbers between each strain and the reference sample for each indicator was calculated using DPS software. A smaller sum indicated a higher comprehensive value for the treatment group. The comprehensive ranking was E > F > G > D > C > B > A > H. Therefore, *C. tropicalis*, *S. cerevisiae*, and *E. faecalis* were selected for subsequent fermentation screening of the compound probiotics.

**Table 5:** Similarity to reference sample and nutritional value ranking of different microbial fermentations.

Strain	SP(% CP)	CP(% DM)	MCP(% DM)	NH_3_-N(g/kg·TN)	pH	Similarity score	Ranking
Reference sample	33.92	16.94	36.66	1.417	4.07	/	/
A	7	7	5	8	3	30	7
B	5	6	6	6	2	25	6
C	6	1	8	7	1	23	5
D	4	3	4	3	7	21	4
E	3	2	2	2	6	15	1
F	2	4	1	4	5	16	2
G	1	5	3	5	4	18	3
H	8	8	7	1	8	32	8

Groups A–G represent fermentation with *L. johnsonii*, *S. alactolyticus*, S. fibuligera, S. boulardii, *C. tropicalis, S. cerevisiae*, and *E. faecalis*, respectively; group H is the blank control. Values in columns SP, CP, MCP, NH_3_-N, and pH are similarity sequence ranks (1 = highest similarity to reference sample, 8 = lowest). Similarity score = sum of ranks across all five indicators; a lower score indicates higher comprehensive value. SP: soluble protein; CP: crude protein; MCP: microbial crude protein; NH_3_-N: ammonia nitrogen.

### 3.2 Screening results of compound probiotics

The L_4_ (16) orthogonal experimental design was adopted, using SP, CP, MCP, NH_3_-N, and pH as indicators to analyze the effects of three strains (a, b, c) on fermentation. The results are shown in Tables 6–10.

**Table 6:** Orthogonal analysis table of SP (% CP).

NO.	Factors			SP(% CP)
	a	b	c	
I	0	0	0	28.83
II	0	1	1	32.84
III	1	0	1	33.96
IV	1	1	0	34.28
SP(% CP)				
K1	30.84	31.40	31.56	
K2	34.12	33.56	33.40	
R	3.29	2.17	1.85	

Factors a, b, and c represent *C. tropicalis*, *E. faecalis*, and *S. cerevisiae*, respectively. “0” indicates the absence of the strain; “1” indicates the presence of the strain. K_1_ and K_2_ represent the mean of the indicator values at the two levels, respectively; *R* (range) = |K_1_ − K_2_|, where a larger *R* indicates a greater influence of that factor on the indicator. Values are means of three replicates. The following tables are presented in the same format.

**Table 7:** Orthogonal analysis table of CP.

NO.	Factors			CP (% DM)
	a	b	c	
I	0	0	0	15.71
II	0	1	1	18.73
III	1	0	1	18.34
IV	1	1	0	19.40
CP (% DM)				
K1	17.22	17.03	17.56	
K2	18.87	19.05	18.54	
R	1.65	2.04	0.98	

**Table 8:** Orthogonal analysis table of MCP.

NO.	Factors			MCP (% DM)
	a	b	c	
I	0	0	0	25.86
II	0	1	1	31.97
III	1	0	1	35.43
IV	1	1	0	43.29
MCP (% DM)				
K1	28.92	30.65	34.58	
K2	39.36	37.63	33.7	
R	10.45	6.99	0.88	

**Table 9:** Orthogonal analysis table of NH_3_-N.

NO.	Factors			NH_3_-N(g/kg TN)
	*a*	*b*	*c*	
I	0	0	0	1.42
II	0	1	1	1.35
III	1	0	1	1.32
IV	1	1	0	1.26
NH_3_-N(g/kg TN)				
K1	1.39	1.37	1.34	
K2	1.29	1.31	1.34	
R	0.1	0.07	0.01	

**Table 10:** Orthogonal analysis table of pH.

NO.	Factors			pH
	*a*	*b*	*c*	
I	0	0	0	6.15
II	0	1	1	4.25
III	1	0	1	5.47
IV	1	1	0	4.19
pH				
K1	5.2	5.81	5.17	
K2	4.83	4.22	4.86	
R	0.37	1.59	0.31	

According to the range analysis, the order of influence of each factor on each indicator and the optimal combinations are presented in Table 11. Among all treatments, Group IV exhibited the highest SP (% CP), CP (% DM), and MCP (% DM), reaching 34.28%, 19.4%, and 43.29%, respectively; Group IV also showed the lowest NH_3_-N (g/kg·TN) and pH, at 1.26 and 4.19, respectively. Overall, factor a had the greatest influence on SP (% CP), MCP (% DM), and NH_3_-N; factor b had the greatest influence on CP (% DM) and pH; and factor c had the least influence on all indicators. According to the range analysis results of each indicator, the formulation scheme of Group IV (containing *C. tropicalis* and *E. faecalis*) was ultimately determined as the superior scheme.

**Table 11:** Summary of orthogonal analysis results for each indicator.

Item	Factor priority order	Superior combination
SP (% CP)	a > b > c	a_1_b_1_c_0_
CP (% DM)	b > a > c	a_1_b_1_c_0_
MCP (% DM)	a > b > c	a_1_b_1_c_0_
NH_3_-N (g/kg·TN)	a > b > c	a_1_b_1_c_0_
pH	b > a > c	a_1_b_1_c_0_

Factor priority order is determined by the range (R) value: a larger R indicates greater influence. The superior combination (a_1_b_1_c_0_) was selected because Group IV achieved the best measured values across all five indicators (for SP, CP, and MCP, higher values are preferred; for NH_3_-N and pH, lower values are preferred). a = *C. tropicalis*, b = *E. faecalis*, c = *S. cerevisiae*; subscript 1 = strain added, 0 = strain not added. SP: soluble protein; CP: crude protein; MCP: microbial crude protein; NH_3_-N: ammonia nitrogen.

### 3.3 Fermentation parameters of the culture medium

As shown in Figure 2A, the pH of the culture medium for substrates with different protein structures gradually decreased over time. However, the differences between treatments and the interaction between treatment and time were not significant (*p* > 0.05), whereas the differences among sampling times were significant (*p* < 0.01). In Figure 2B, both sampling time and treatment had significant effects on NH_3_-N concentration (*p* < 0.05), while the interaction between time and treatment was not significant (*p* > 0.05). Groups S1 and S3 maintained higher NH_3_-N levels during the 6–48 h period. As shown in Figure 2C, the GP value of the culture medium increased gradually over time, with both time and treatment having highly significant effects (*p* < 0.01), and no significant differences in their interaction (*p* > 0.05).

**Figure 2:**
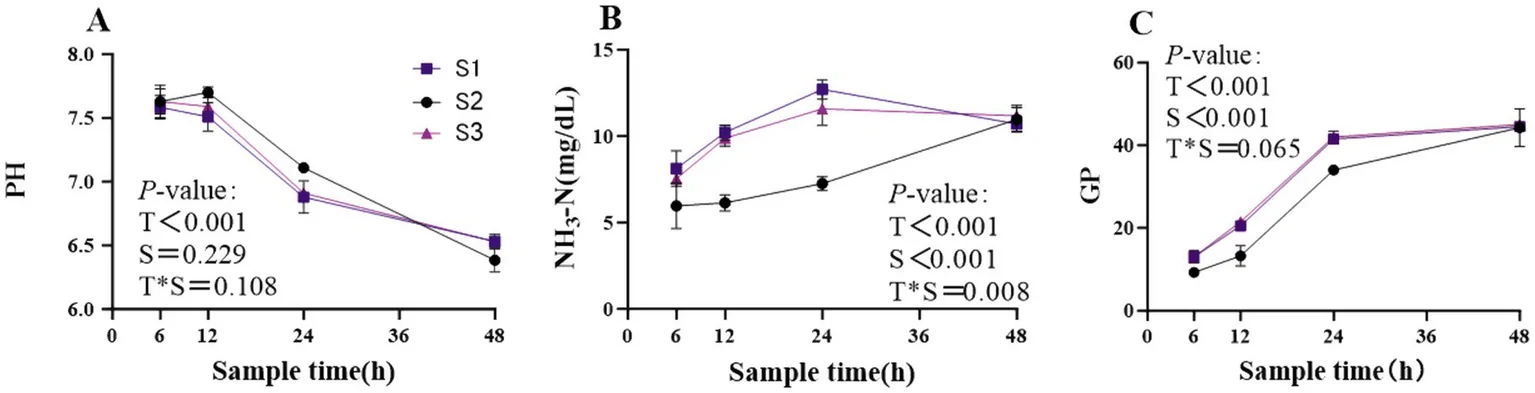
Changes in pH **(A)**, ammonia nitrogen (NH_3_-N) concentration **(B)**, and gas production (GP) **(C)** at different cultivation time points (*n* = 6). S1, S2, and S3 represent substrates with SP:ISP ratios of 1:2, 1:2.5, and 1:3, respectively, all fermented with the compound probiotic (*C. tropicalis* + *E. faecalis*). Data are presented as mean ± SD. A two-way ANOVA was used to evaluate the effects of time (T), treatment (S), and their interaction (T × S). Asterisks indicate significant differences between groups at the corresponding time point (*p* < 0.05).

As shown in Table 12, significant differences were observed in the concentrations of TVFA, acetate, propionate, and valerate among the different groups (*p* < 0.05). The concentration of isovalerate in the S1 group was significantly lower (*p* < 0.05), whereas butyrate and isobutyrate did not differ significantly among groups (*p* > 0.05).

**Table 12:** Volatile fatty acid content in the culture medium of each group.

Item	Treatment			SEM	*p*-Value
	S1	S2	S3		
TVFA	103.08^c^	144.08^a^	116.15^b^	9.955	0.031
Acetate	54.68^c^	62.85^a^	57.83^b^	2.147	0.015
Propionate	21.99^c^	25.78^a^	23.93^b^	1.096	0.030
Butyrate	8.41	9.40	9.87	0.562	0.143
Isobutyrate	1.45	6.69	4.11	2.118	0.251
Valerate	2.57^c^	11.57^a^	7.04^b^	2.316	0.045
Isovalerate	6.97^b^	20.78^a^	15.37^a^	3.432	0.039
Acetate/Propionate Ratio	2.46	2.49	2.40	0.054	0.316

S1, S2, and S3 represent substrates with SP:ISP ratios of 1:2, 1:2.5, and 1:3, respectively, all fermented with the compound probiotic. Values are means (*n* = 6), with pooled standard error of the mean (SEM). VFA concentrations are expressed in mmol/L. Differences among groups were analyzed by one-way ANOVA followed by Duncan’s multiple-range test. Within a row, means sharing the same letter or bearing no letter do not differ significantly (p > 0.05); means with different letters differ significantly (p < 0.05). TVFA: total volatile fatty acids.

### 3.4 Substrate degradation efficiency

As shown in Figure 3A, the CPD of S1 and S2 was significantly higher than that of S3 (*p* < 0.05), with S1 and S2 not differing significantly from each other. As shown in Figure 3B, both sampling time and interaction between time and treatment significantly affected the DMD (*p* < 0.05), whereas the differences among the various treatments were not significant (*p* > 0.05). At the end of the fermentation period, DMD was numerically highest in S2, followed by S3 and S1.

**Figure 3:**
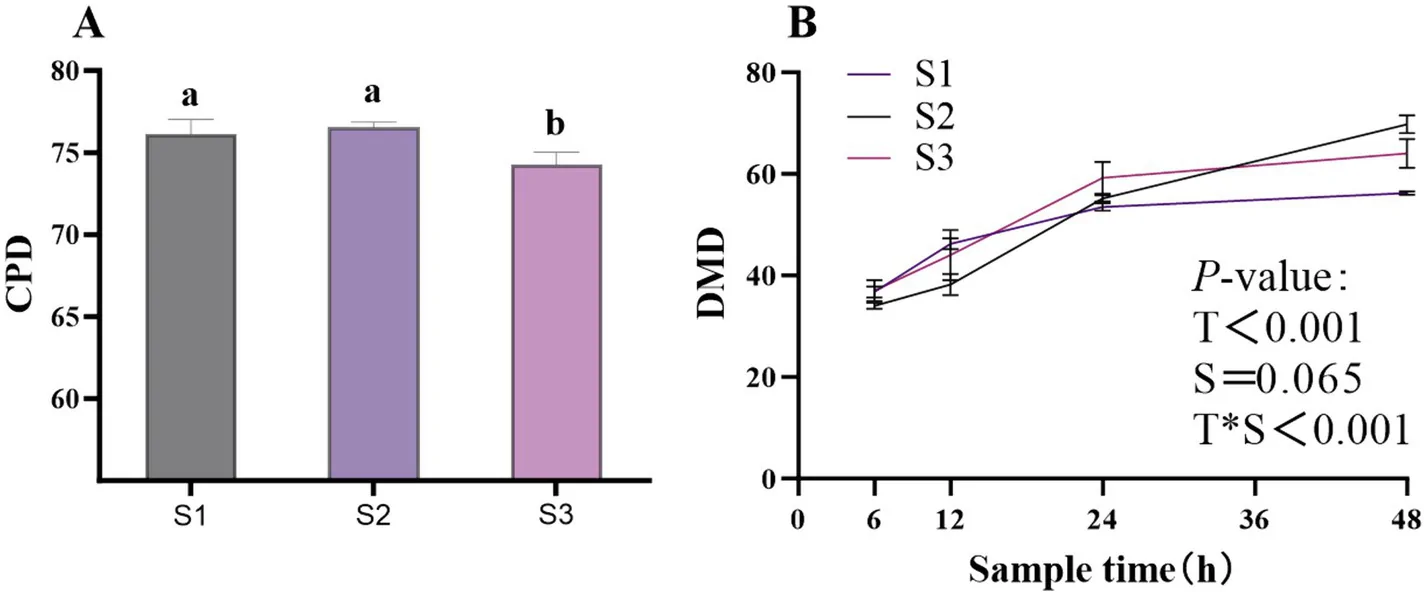
Substrate crude protein disappearance (CPD) at the fermentation endpoint **(A)** and dry matter disappearance (DMD) over fermentation time **(B)** (*n* = 6). S1, S2, and S3 represent substrates with SP:ISP ratios of 1:2, 1:2.5, and 1:3, respectively, all fermented with the compound probiotic. Data are presented as mean ± SD. For endpoint CPD **(A)**, differences among groups were analyzed by one-way ANOVA followed by Duncan’s multiple-range test; different lowercase letters indicate significant differences (*p* < 0.05). For DMD **(B)**, a two-way ANOVA was used to evaluate the effects of time (T), treatment (S), and their interaction (T × S).

### 3.5 Rumen microbiota

The 16S rRNA gene sequencing results indicated that optimization of the substrate protein structure modulated the rumen microbial community composition (Figure 4). The Chao, Ace, and Simpson indices (reflecting microbial richness) showed no significant differences among the treatment groups (*p* > 0.05; Figure 4A), suggesting that changes in substrate protein structure did not alter the richness of the rumen microbial community. However, a significant difference in the Shannon index was observed between the S1 and S3 groups (*p* < 0.05). Venn diagram analysis revealed that, at the operational taxonomic unit (OTU) level, the S1, S2, and S3 groups contained 233, 159, and 217 unique OTUs, respectively (Figure 4B). Principal coordinate analysis (PCoA) demonstrated distinct differences in the microbial community structure (beta diversity) among the groups (*p* < 0.05; Figure 4C). The rarefaction curves plateaued toward the end, indicating sufficient sequencing depth and reliable data (Figure 4D).

**Figure 4:**
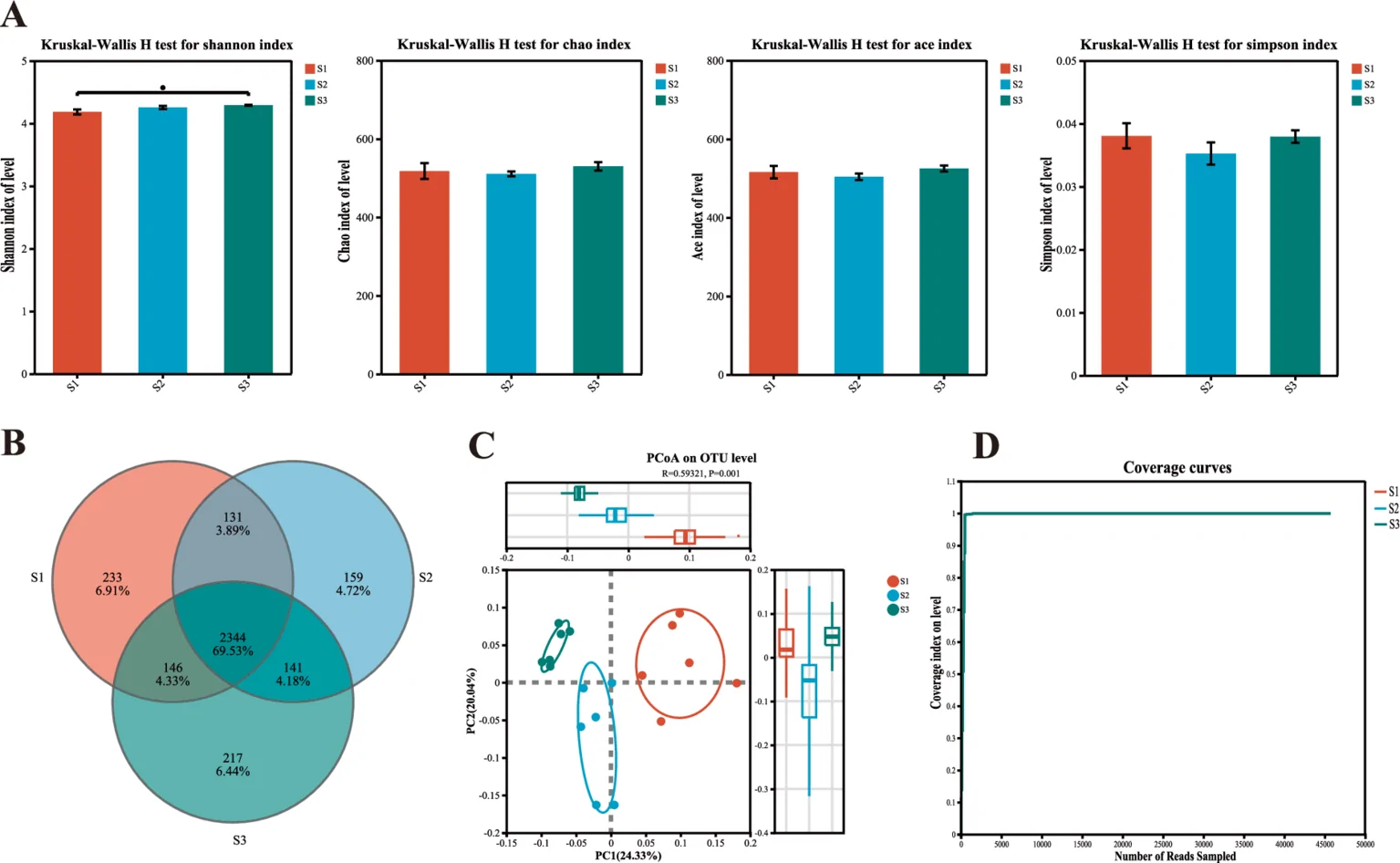
Composition of the rumen microbial community in the *in vitro* culture medium (*n* = 6). **(A)** α-Diversity indices (Shannon, Chao, Ace and Simpson) evaluating microbial richness and evenness. **(B)** Venn diagram depicting the distribution of exclusive and common operational taxonomic units (OTUs) across experimental cohorts. **(C)** Principal coordinates analysis (PCoA) plot visualizing β-diversity and inter-group dissimilarity. **(D)** Rarefaction curves indicating sequencing depth adequacy. S1, S2, and S3 represent substrates with SP:ISP ratios of 1:2, 1:2.5, and 1:3, respectively. Significance is indicated by asterisks: *p < 0.05, **p < 0.01 (FDR-adjusted).

At the genus level, *Rikenellaceae_RC9_gut_group*, n*orank_f_F082*, and *Xylanibacter* were the dominant genera (Figure 5A). At the phylum level, *Bacteroidetes* and *Bacillota* were the two dominant phyla, collectively accounting for > 83% of the total 16S rRNA gene sequences (Figure 5B). Comparison of the rumen microbial community composition among the three substrate groups showed that the relative abundances of *Streptococcus* and *Ruminobacter* were significantly higher in the S2 group (*p* < 0.05), whereas the relative abundance of R*ikenellaceae_RC9_gut_group* was significantly lower in the S2 and S3 groups than in the S1 group (*p* < 0.05, Figure 5C). The relative abundance of *Pseudomonadota* was significantly higher in the S1 group compared to the other two groups (*p* < 0.05), whereas the relative abundance of *Bacteroidetes* was significantly higher in the S2 group than in S1 and S3 (*p* < 0.05; Figure 5D). Linear discriminant analysis (LDA) effect size (LEfSe) analysis was performed on the core microbiota, revealing the following treatment-specific enrichments: the *o-Enterobacterales*, *f-Succinivibrionaceae*, and *p-Pseudomonadota* were specifically enriched in the S1 group; the *o-Bacteroidales*, *c-Bacteroidia*, and *p-Bacteroidota* were specifically enriched in the S2 group; and the *f-Rikenellaceae* was specifically enriched in the S3 group (Figure 5E).

**Figure 5:**
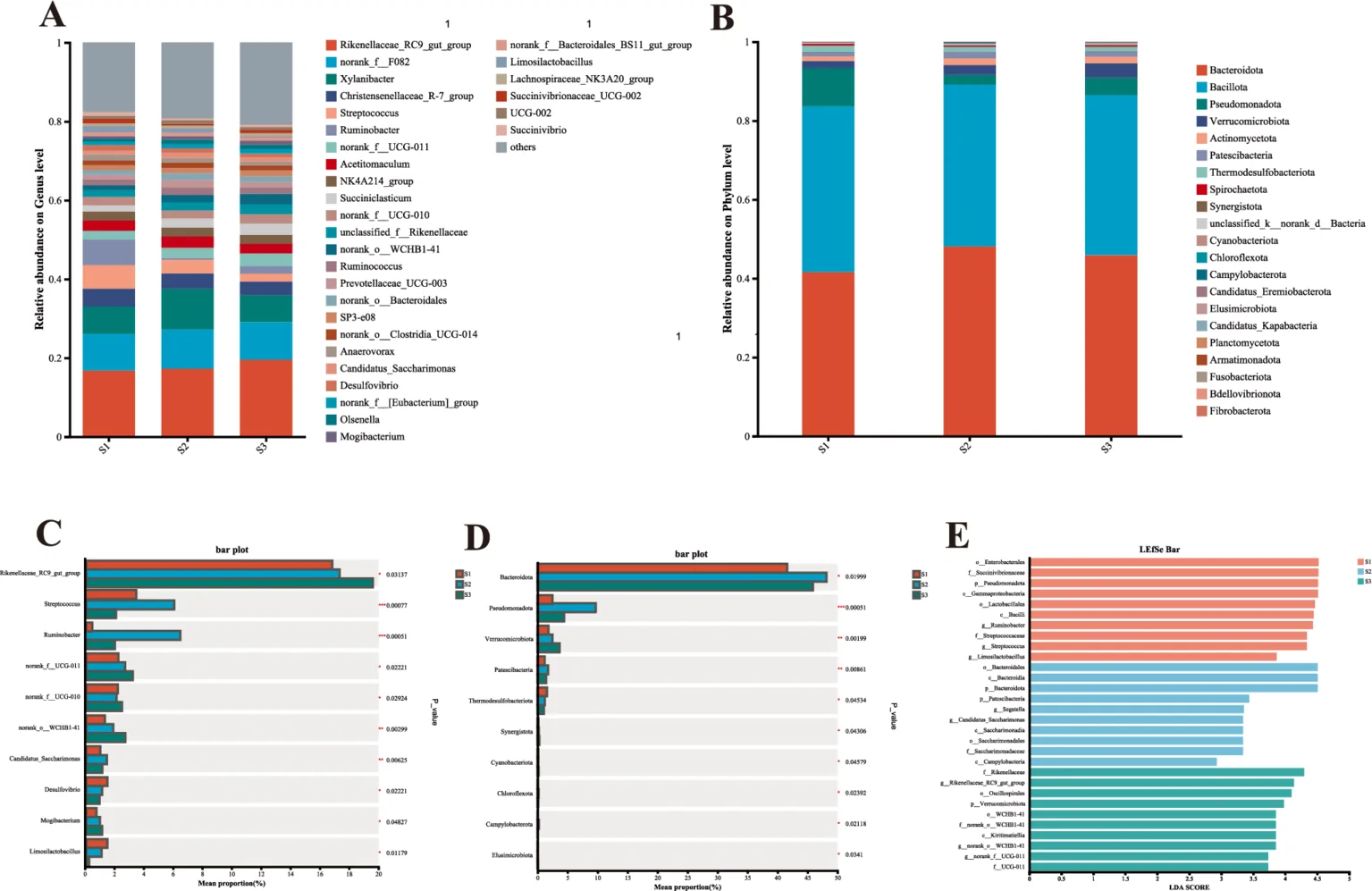
Composition of the rumen microbial community in the *in vitro* culture medium (n = 6). **(A)** Genus-level community composition. **(B)** Phylum-level community composition. **(C)** Differential abundance analysis showing significant differences at the Genus level among groups. **(D)** Differential abundance analysis showing significant differences at the Phylum level among groups. **(E)** Linear discriminant analysis (LDA) coupled with effect size (LEfSe) identifying differentially abundant microbial taxa across groups. S1, S2, and S3 represent substrates with SP:ISP ratios of 1:2, 1:2.5, and 1:3, respectively. Significance is indicated by asterisks: *p < 0.05, **p < 0.01, ***p < 0.001 (FDR-adjusted).

## 4 Discussion

### 4.1 Effects of single-strain fermentation on feed

Solid-state fermentation of feedstuffs is a complex and multidimensional process. The effect of single-strain fermentation on the substrate is primarily reflected in the increased proportion of SP, which provides a more readily available nitrogen source for subsequent ruminal utilization. The fermentation process not only alters the nutritional composition of the feed but also, by influencing the rumen microbiota and metabolic activities, subsequently optimizes the digestive absorption efficiency and production performance of the animals. The 48-h fermentation was employed as a pretreatment step to modify the substrate protein profile prior to rumen incubation, rather than reflecting rumen degradation kinetics. In the subsequent *in vitro* rumen fermentation, SP (particularly the PA and PB1 fractions in the CNCPS system) is characterized by a rapid degradation rate in the rumen and serves as a readily available nitrogen source for rumen microorganisms. Regulating the SP level is of great significance for improving nitrogen utilization efficiency in ruminants. Fermentation generally increases the SP content in feed because microbial proteases hydrolyze macromolecular proteins in the feed, making them more readily available to rumen microorganisms (21). In this experiment, after the substrates were fermented with probiotics, the SP content in all experimental groups was significantly increased compared with the control group, indicating that most probiotics possess the ability to increase soluble protein content, albeit with varying degrees of effectiveness. In the diet of dairy cows during peak lactation, the SP level in the protein component accounts for 30–35% of CP (18). If this proportion is too high, it can lead to an increased transient release peak of ammonia, decreased nitrogen utilization efficiency, and subsequent excretion in the form of urea. Among the various groups, *C. tropicalis*, *S. cerevisiae*, and *E. faecalis* demonstrated a significantly greater ability to increase the substrate’s SP content than the other strains, with the content falling within the 30–35% range.

Crude protein is a measure of the total nitrogen content in feed or biomass. Fractionation of protein-rich coproducts such as distillers dried grains with solubles using the Cornell Net Carbohydrate and Protein System has shown that a considerable proportion of their protein is present as soluble fractions (22). In this experiment, as the SP content increased, the CP content also correspondingly increased, consistent with the above fractionation pattern. Of the protein absorbed in the small intestine of ruminants, 60–85% of the nitrogen is derived from rumen-synthesized MCP (23). Increased dietary SP levels can, within a certain range, promote MCP synthesis (16). However, when the SP level exceeded 40%, the transient ammonia release peak surpassed the microbial capture capacity, leading to a decrease in MCP synthesis efficiency, which manifested as increased milk urea nitrogen and decreased true protein (24). In this study, groups E, F, and G showed significantly elevated SP levels in the substrate. Concurrently, the MCP content also showed a significant increase compared to that in the other groups, consistent with the mechanistic description in the aforementioned research. In the rumen, SP is rapidly hydrolyzed to release ammonia nitrogen, which serves as the primary nitrogen source for microbial protein synthesis (17). Similarly, during solid-state fermentation, microbial proteolysis also generates peptides, amino acids, and ammonia. In the present experiment, however, groups in which the substrate SP level increased after fermentation showed a significant decrease in the corresponding NH_3_-N content. This could be attributed to microbial assimilation. When a system’s carbon-to-nitrogen (C/N) ratio is appropriate or carbon sources are sufficient, microorganisms preferentially assimilate NH_3_-N for growth and MCP synthesis, a key pathway of microbial nitrogen assimilation (25).

pH serves as an indicator of microbial activity. An increase or decrease in its value can effectively indicate whether the target strain is in its optimal physiological state, and whether the system has been effectively dominated by that strain, thereby suppressing undesirable microbes. In a study investigating the fermentation performance of nine lactic acid bacterial strains with probiotic potential during sorghum fermentation, all strains effectively reduced the pH of the medium to below 4.0 within 24 h, demonstrating the potential for improving the safety and quality of fermented products (26). In the present study, the pH of the inoculated substrates dropped to approximately 4.0–4.2, with no visible mold or off-odor, indicating good fermentation quality. This was consistent with the findings of previous research. The pH adaptability of yeast during alcohol fermentation has also been extensively studied. Research shows that the optimal growth temperature and pH range for *Saccharomyces cerevisiae* are 20–40 °C and 3.0–8.0, respectively (27). In this study, the final pH value from single-strain yeast fermentation was within the normal range.

### 4.2 Effects of compound probiotic fermentation on feed

During the screening stage, the compound probiotic combination outperformed the single strains in improving substrate quality, as reflected in higher SP and MCP contents and lower NH_3_-N and pH. This is likely due to the fact that during the initial fermentation stage, yeasts, as facultative anaerobes, rapidly consume the residual oxygen in the system through respiration. This creates the necessary conditions for growth and reproduction of lactic acid bacteria, thereby improving overall fermentation quality (28). A higher SP content indicates stronger nitrogen source availability. A higher MCP content suggests strong microbial activity and high nitrogen utilization efficiency, while an increase in NH_3_-N concentration typically leads to nitrogen loss and reduced efficiency of microbial nitrogen conversion. This study confirmed that the coculture system of *C. tropicalis* and *E. faecalis* exhibited a significant synergistic effect in enhancing the soluble protein content of the substrate. This finding was consistent with recent research trends on fungal-bacterial cross-kingdom interactions regulating microenvironmental metabolism, indicating that the two microorganisms do not act independently but rather form a functionally complementary community through complex metabolic coupling. The molecular basis of the synergistic effect primarily stems from a complementary proteolytic network. The highly active extracellular proteases secreted by *E. faecalis* (29) initiate protein degradation in the initially neutral environment, whereas the aspartic proteases secreted by *C. tropicalis* (30) further cleave large peptide fragments into small peptides and amino acids. Furthermore, the organic acids produced by the metabolism of *E. faecalis* reduce the microenvironmental pH, which not only inhibits competing spoilage bacteria but may also increase protein solubility by altering the charge state of proteins, thereby creating favorable conditions for subsequent enzymatic hydrolysis. The observed improvement in nitrogen utilization efficiency can be attributed to the metabolic cross-feeding mechanism between the two microorganisms, whereby *C. tropicalis* releases free amino acids by degrading complex proteins and secretes essential growth factors such as B vitamins, thereby relieving the nutritional limitation of *E. faecalis*; such nitrogen-overflow-mediated cross-feeding from yeasts to lactic acid bacteria has been documented previously (31). Subsequently, *E. faecalis* efficiently assimilates these nitrogen sources for microbial protein synthesis, achieving the rapid conversion of nitrogen from the non-protein form to the microbial protein form. This tight “degradation–assimilation” coupling reduces ammonia nitrogen volatilization loss and significantly enhances the nitrogen retention rate within the system. This synergistic effect is not limited to *in vitro* fermentation but also holds potential regulatory value for the *in vivo* rumen or intestinal microecology. By providing a high proportion of soluble peptides, this combination optimizes the substrate supply profile for rumen microorganisms, promotes the proliferation of beneficial microbial populations, and improves the nitrogen metabolism balance of the host.

Although this study revealed the synergistic effect at the phenotypic level, the specific molecular signaling pathways and gene expression regulatory networks still need to be further elucidated through transcriptomics and metabolomics. Future research should focus on the immediate effects of the interaction between the two microorganisms on the expression of host immune receptors and specific nitrogen transporters under dynamic conditions, in order to quantify their exact contribution in complex ecosystems.

### 4.3 Impact on fermentation parameters of the culture medium

The pH and NH_3_-N concentration in the *in vitro* culture medium are significant indicators reflecting rumen fermentation. The normal rumen fluid pH is generally considered to be within the range of 6.0–7.4, whereas during rumen acidosis the pH can drop below 5.4, directly impairing nutrient utilization and threatening animal health (32). In this study, the pH of the culture medium for substrates with different protein structures gradually decreased over time, fluctuating within the range of 6.47–7.64—mostly within the normal physiological range—with no significant differences among treatments. Furthermore, there was no significant difference in the endpoint pH, which was consistent with previous *in vitro* observations (17). Changes in rumen fluid NH_3_-N concentration reflect both the degradation rate of dietary nitrogen by rumen microbes and the utilization rate of NH_3_-N by these microbes. When the NH_3_-N concentration is between 5.0 and 30.0 mg/dL, microbial GP and MCP synthesis peak simultaneously. Concentrations below 5 mg/dL or above 35 mg/dL significantly inhibit fermentation (33, 34). In this study, the NH_3_-N concentration in the culture medium across different substrate protein structure treatments was controlled within the range of 5.0–13.1 mg/dL, all falling within the normal concentration range. However, differences in substrate protein structure significantly altered the NH_3_-N concentration in the culture medium. The NH_3_-N concentration in the S1 group remained at a relatively high level between 12 and 24 h, which is related to the higher SP level in its substrate. Large amounts of SP in the substrate rapidly decompose in the rumen, generating ammonia, peptides, and amino acids. When ruminal microbes cannot utilize these products simultaneously, NH_3_-N accumulates. This finding was consistent with the results reported by Zhang et al. (17).

In ruminant nutrition, the level of VFA in rumen fluid is a critical parameter for assessing rumen fermentation. VFA are produced by the rumen microbial degradation of dietary carbohydrates. Subsequently, VFA (especially propionate) are absorbed through the rumen wall and transported to the liver, where they are further converted into nutrients, such as fats and glycogen, that the body can directly utilize (35). This constitutes a unique energy-supply pathway for ruminants. In this *in vitro* experiment, the levels of TVFA, acetate, propionate, and valerate in the culture medium were significantly different due to variations in the substrate protein structure. Differences in substrate SP (% CP), without considering the synchronized release of energy and nitrogen, may disrupt the energy-nitrogen balance when the substrate SP level is either too low or too high. This can result in reduced acid production during fermentation, which explains the lower VFA content observed in groups S1 and S3 in this experiment. Conversely, the significantly higher VFA content in Group S2 was likely attributable to a more optimal SP (% CP) ratio in the substrate. The acetate-to-propionate molar ratio is used to evaluate microbial fermentation patterns. In this study, the differences in the acetate-to-propionate ratio among the treatment groups were not significant, indicating that the different substrate protein structures did not alter the rumen fermentation pattern, which remained inclined toward acetate fermentation. This finding was consistent with the results reported by Ma et al. (36).

### 4.4 Impact on substrate degradation efficiency

The metabolic extent of substrate nutrients shows a significant positive correlation with rumen microbial activity and is widely used as a reliable indicator of fermentation depth (37). In this study, the DMD increased over time and gradually plateaued after 24 h. DMD showed a significant time × treatment interaction but no significant treatment main effect; at the 48-h endpoint, DMD was numerically highest in S2, followed by S3 and S1. This indicates that both excessively low and excessively high substrate SP levels may adversely affect the fermentation and utilization efficiency of the substrate, which consistent with the findings of Zhang et al. (17), who reported reduced dry matter and nitrogen digestibility at SP levels of both 20 and 50% of CP. Furthermore, CPD was significantly higher in S1 and S2 than in S3. These changes in the degree of fermentation are likely closely related to the quantity and structure of the microbial population in the fermentation medium.

### 4.5 Impact on the rumen microbial community

Rumen microorganisms play crucial roles in ruminant nutrition. Their core function is to degrade various compounds in the feed, thereby supplying essential nutrients to the host (38). Within the rumen, a large number of microbes participate in the degradation of dietary protein, and rumen bacteria play a primary role in this process (39). Dietary manipulation not only drives changes in the alpha and beta diversity of rumen microbial communities but may also, through these changes, influence feed utilization efficiency in ruminants (40). This study employed 16S rRNA sequencing to analyze the effects of different substrate protein structures on rumen bacterial community diversity in beef cattle, including alpha diversity indices reflecting species richness and evenness. The Chao and Ace indices, which measure species richness, were not affected by changes in substrate protein structure. The Simpson index, which is more sensitive to the dominant species, was also unaffected. However, a significant difference in the Shannon index was observed between groups S1 and S3. This suggests that overall richness and broad diversity were generally consistent across the groups during the experimental period. The difference in the Shannon index, which is sensitive to community evenness, might be attributed to the relative enrichment of a few high-abundance OTUs or specific genera in the S3 group; such enrichment can reduce community evenness and lower the Shannon value while having minimal impact on indices such as Chao, Ace, and Simpson. This interpretation was consistent with the established properties of alpha-diversity indices (41). Beta diversity analysis revealed that the rumen bacterial communities of the three groups clustered separately, indicating that the bacterial community structure and composition were influenced by different substrate protein structures. The plateauing of rarefaction curves suggests sufficient sequencing depth and reliable data. The predominant protein-degrading bacteria in the rumen include *Bacteroides* spp., *Prevotella* spp., *Ruminobacter amylophilus*, *Clostridium* spp., *Butyrivibrio fibrisolvens*, *Eubacterium ruminantium*, *Streptococcus bovis*, among others. Among these, *R. amylophilus*, *B. fibrisolvens*, *Prevotella* spp., and *S. bovis* play major roles in the degradation of SP in the rumen and can influence degradation rates and nitrogen loss as ammonia (42). In this experiment, different substrate protein structures significantly affected the relative abundance of *Streptococcus* and *Ruminobacter* in the culture medium, with the highest relative abundances observed in the S2 group. Notably, both genera include species that have been implicated in ruminal protein degradation. Therefore, the increased abundance of these taxa may be associated with, and potentially contribute to, the improved nitrogen utilization observed in the S2 group; however, these findings reflect correlative associations rather than causal relationships. As the present study did not directly measure functional gene expression or nitrogen-metabolism-related enzyme activities, the specific functional contributions of these taxa warrant further investigation in future studies, for instance, through metagenomics, metatranscriptomics, or enzyme activity assays.

### 4.6 Limitations

Several limitations should be acknowledged when interpreting the results.

First, although fermentation conditions (strain composition, inoculation rate, moisture content, temperature, and duration) were identical across groups, a comprehensive compositional analysis of the fermented substrates –particularly carbohydrate fractions (NDF, ADF, starch, and water-soluble carbohydrates)– was not performed. As probiotic fermentation can alter both nitrogen and carbohydrate fractions, the observed differences in VFA production, DMD, CPD, and microbial community structure cannot be attributed solely to the initial SP:ISP ratio. Second, the *in vitro* model, while allowing precise control of variables, lacks the systemic complexity of the *in vivo* environment, including host immune responses, ruminal absorption, and passage kinetics, which limits direct extrapolation to living animals. Third, only a limited number of strains were evaluated; given the strain specificity of probiotic effects, the findings may not be generalizable to other untested strains. Fourth, the absence of animal performance metrics prevents confirmation of whether the observed microbial shifts translate into production benefits. Additionally, rumen fluid was collected from only three donor animals, which may not capture inter-animal variability in microbial communities. Finally, the study relied on taxonomic profiling and basic fermentation parameters, lacking functional analyses such as metagenomics or enzymatic activity assays, which limits elucidation of the underlying metabolic pathways.

Future studies should include post-fermentation compositional profiling and integrate *in vivo* trials with multi-omics approaches to comprehensively validate the efficacy and mechanisms of these probiotic candidates.

## 5 Conclusion

Supplementing the fermentation substrate with *C. tropicalis* and *E. faecalis* significantly increased the proportion of SP in the substrate after fermentation, thereby increasing the proportion of readily available protein in the diet. Furthermore, *in vitro* rumen fluid fermentation simulation experiments indicated that when the substrate protein structure had an SP:ISP ratio of 1:2.5 and fermentation was conducted with the aforementioned compound probiotics, the relative abundance of microorganisms related to nitrogen utilization was elevated, which may contribute to improved nitrogen utilization efficiency under *in vitro* conditions. The findings of this study provide data to support the development of novel microecological feed additives based on local feed resources and offer preliminary guidance for the collaborative regulation of rumen nitrogen metabolism through adjustment of dietary protein structure combined with probiotic supplementation. However, these findings warrant further *in vivo* validation.

## Data Availability

The original contributions presented in the study are publicly available. This data can be found here: Raw sequences were submitted to the NCBI Sequence Read Archive (SRA) (accession number PRJNA1404885).
